# Determinants of polyp Size in patients undergoing screening colonoscopy

**DOI:** 10.1186/1471-230X-11-101

**Published:** 2011-09-24

**Authors:** Albert B Lowenfels, J Luke Williams, Jennifer L Holub, Patrick Maisonneuve, David A Lieberman

**Affiliations:** 1 Department of Surgery, New York Medical College, Valhalla, USA; 2 Division of Gastroenterology and Hepatology, Department of Medicine, Oregon Health and Science University, Portland, USA; 3 Division of Epidemiology and Biostatistics, European Institute of Oncology, Milan, Italy; 4 Division of Gastroenterology, Portland VA Medical Center, Portland, USA

**Keywords:** Colorectal, colon, polyps, colonoscopy, cancer, size

## Abstract

**Background:**

Pre-existing polyps, especially large polyps, are known to be the major source for colorectal cancer, but there is limited available information about factors that are associated with polyp size and polyp growth. We aim to determine factors associated with polyp size in different age groups.

**Methods:**

Colonoscopy data were prospectively collected from 67 adult gastrointestinal practice sites in the United States between 2002 and 2007 using a computer-generated endoscopic report form. Data were transmitted to and stored in a central data repository, where all asymptomatic white (n = 78352) and black (n = 4289) patients who had a polyp finding on screening colonoscopy were identified. Univariate and multivariate analysis of age, gender, performance site, race, polyp location, number of polyps, and family history as risk factors associated with the size of the largest polyp detected at colonoscopy.

**Results:**

In both genders, size of the largest polyp increased progressively with age in all age groups (*P *< .0001). In subjects ≥ 80 years the relative risk was 1.55 (95% CI, 1.35-1.79) compared to subjects in the youngest age group. With the exception of family history, all study variables were significantly associated with polyp size (*P *< .0001), with multiple polyps (≥ 2 versus 1) having the strongest risk: 3.41 (95% CI, 3.29-3.54).

**Conclusions:**

In both genders there is a significant increase in polyp size detected during screening colonoscopy with increasing age. Important additional risk factors associated with increasing polyp size are gender, race, polyp location, and number of polyps, with polyp multiplicity being the strongest risk factor. Previous family history of bowel cancer was not a risk factor.

## Background

Globally, colorectal cancer is now the fourth commonest tumor in males and the third most frequent tumor in females[1]. Numerous studies have confirmed the strong relationship between colorectal polyps and colorectal cancer and there is universal agreement that pre-existing polyps are the major risk factor leading to the subsequent development of colorectal cancer[2]. Polyps with advanced histological features are especially likely to be premalignant[3].

It has been well documented that the proportion of polyps harboring advanced histologic features increases with the polyp size. For example, polyps less than 5 mm rarely have worrisome pathologic features, in contrast to polyps ≥ 10 mm where about one third will have advanced histology[4-6].

Information about determinants of polyp size could be potentially informative for recommendations about the age of onset of initial screening, the interval between screening procedures, and the type of screening procedure to be employed.

However, despite the strong link between polyp size and the subsequent development of malignancy, relatively few studies have investigated age-related changes in polyp size or have investigated risk factors leading to the growth of polyps[5-7]. The main reason for the paucity of information on polyp growth is that such studies generally require repeated observations on polyps that have been left in situ, rather than being removed. However, since even small polyps can be occasionally malignant, observing rather than resecting polyps leads to ethical problems. Those rare studies that have been performed generally have contained only a few patients, thus limiting their inferential power. Some have been performed almost 50 years ago and were based on indirect repeated measurements of colorectal polyps observed by serial barium enemas.

To obtain quantitative information about risk factors that determine polyp size we have used stored observational data on polyp size from a large database of patients undergoing screening colonoscopy. The primary study aim was to determine risk factors that are related to polyp size in males and females. We hypothesized that in addition to previously determined factors such as age and race, other factors such as family history of colorectal cancer, gender, anatomic location of the polyp, and number of polyps might be important predictors of polyp size.

## Methods

### Data source

The data included in this report were obtained from a consortium of 535 physicians practicing at 67 practice sites in 26 states, who participate in the Clinical Outcomes Research Initiative (CORI) using previously described methodology[8,9]. Briefly, all cooperating centers used the same computerized form to transmit data obtained from colonoscopic procedures to a central registry where the data are subjected to quality control and then merged. The CORI database is a limited dataset, and does not contain patient contact information such as name or address. This project was approved by the Oregon Health & Science University institutional review board.

### Patients

Patients came from three separate sources: private practice (79%), academic sites (10%), and patients treated by the Department of Veterans Affairs (11%). Patients underwent screening colonoscopy in both urban and rural sites.

We collected data from patients ≥ 18 years undergoing first colonoscopy at a CORI-participating site during the study period without prior symptoms or signs such as pain or bleeding, and without known colorectal cancer syndromes during the period 2002 to 2007. Patients diagnosed with polyps during the procedure were considered for inclusion in the study. From the available colonoscopy patient database we excluded records of patients missing data about polyp size (n = 3899) or location (n = 4103), and an additional 213 patients with polyps > 40 mm, leaving 82641 patients for analysis.

Mandatory variables included on the initial data collection form and used for this study were: age, gender, race (black non-Hispanic, white non-Hispanic), type of practice, size of the largest polyp detected at colonoscopy, number of polyps (single versus ≥ 2), any family history of colorectal cancer (positive, negative) and polyp location defined as proximal or distal to the splenic flexure. Histologic results were available for some patients, but no information about any type of histologic finding was included in the present study because only approximately 25% of CORI sites enter pathology data. Classification of race (White or Black) was provided by the endoscopist; these two categories accounted for > 90% of all patients in the database.

### Statistical analysis

Patient age was stratified by decade with additional categories for patients < 50 years and ≥ 80 years. For univariate analysis of mean polyp size and 95% confidence limits, we used chi-square tests and t-tests to study age-related size differences in subgroups stratified by gender, race, polyp location, and number of polyps.

We used ordinal logistic regression to obtain multivariate adjusted estimates with 95% confidence intervals. Ordinal logistic regression is a proportional odds model that allowed us to generate odds ratios measuring the risk associated with an increase of polyp size corresponding to moving from one category to the next larger size category. For this analysis we stratified polyps by size into four groups: 0-5 mm, 5.1-9.0 mm, 9.1-15 mm, and 15-40 mm. The method assumes similar odds ratios associated with transition from one group to the next larger group. We tested this assumption, which proved to be accurate for our data set, allowing us to provide a common odds ratio for each of the variables studied. Variables included in the multivariate adjusted model included age, sex, race, number of polyps, location of polyp (proximal or distal), procedure site. Any family history of colorectal cancer was included as a variable because it has been previously reported to be a predictor of polyp growth[10,11]. Because of a statistically significant interaction between gender and other variables, we present separate multivariate-adjusted data for males and females. To insure inclusion of all patients with polyps in the study we excluded polyps > 40 mm on the assumption that lesions > 40 mm were unlikely to be polyps. All analytic procedures were carried out with SAS (version 9.1; SAS Institute Inc, Cary, North Carolina).

## Results

Table 1 provides information about patient (n = 82641) and polyp characteristics stratified by the size of the largest detected polyp. The mean age of all patients was 60.9 ± 9.2 years of age. Forty one percent of patients were females and Blacks represented 5.2% of the total group. Almost 80% of colonoscopies were performed in community or HMO settings; polyps detected in patients undergoing colonoscopy in these types of practices were larger than in either academic or VA or military sites (Table 1). In the entire data set more than half of all polyps were small, measuring ≤ 5 mm; polyps 9-15 mm represented about 12-15% of the entire group, and polyps > 15 mm were rare, constituting < 5% of all polyps. With increasing age, smaller polyps became less common, while larger polyps increased in frequency. Approximately two-thirds of patients had a single polyp and family history of colorectal cancer was reported to be negative in three-fourths of patients.

**Table 1:** Patient demographics by polyp size

	Polyp Size								
	0 - 5 mm		> 5 - 9 mm		> 9 - 15 mm		> 15 mm		Total
	n	(%)	n	(%)	N	(%)	n	(%)	n
**All Subjects**	48947	(59.2)	19422	(23.5)	11240	(13.6)	3032	( 3.7)	82641
**Age**									
< 50	3097	(63.4)	1073	(22.0)	587	(12.0)	125	( 2.6)	4882
50-59	22859	(61.8)	8433	(22.8)	4613	(12.5)	1081	( 2.9)	36986
60-69	14913	(57.3)	6259	(24.1)	3809	(14.6)	1035	( 4.0)	26016
70-79	6991	(55.0)	3149	(24.8)	1905	(15.0)	658	( 5.2)	12703
80+	1087	(52.9)	508	(24.7)	326	(15.9)	133	( 6.5)	2054
**Gender**									
Male	28002	(57.9)	11502	(23.8)	6982	(14.4)	1909	( 2.3)	48395
Female	20945	(61.2)	7920	(23.1)	4258	(12.4)	1123	( 3.3)	34246
**Site**									
Academic	4864	(64.4)	1506	(19.9)	928	(12.3)	257	( 3.4)	7555
Community/HMO	38503	(58.4)	15909	(24.1)	9019	(13.7)	2491	( 3.8)	65922
VA/Military	5580	(60.9)	2007	(21.9)	1293	(14.1)	284	( 3.1)	9164
**Race**									
White	46601	(59.5)	18436	(23.5)	10506	(13.4)	2809	( 3.6)	78352
Black	2346	(54.7)	986	(23.0)	734	(17.1)	223	( 5.2)	4289
**Location**									
Proximal Colon	20013	(55.6)	9266	(25.7)	5198	(14.4)	1524	( 4.2)	36001
Distal Colon and, Rectum	28934	(62.0)	10156	(21.8)	6042	(13.0)	1508	( 3.2)	46640
**Number of polyps**									
Single	38027	(69.1)	10676	(19.4)	5057	( 9.2)	1295	( 2.4)	55055
Multiple	10920	(39.6)	8746	(31.7)	6183	(22.4)	1737	( 6.3)	27586
**Family history**									
Negative	37015	(59.1)	14669	(23.4)	8642	(13.8)	2322	( 3.7)	62648
Positive	11932	(59.7)	4753	(23.8)	2598	(13.0)	710	( 3.6)	19993

*Numbers in brackets = percent of row total.

**Figure 1:**
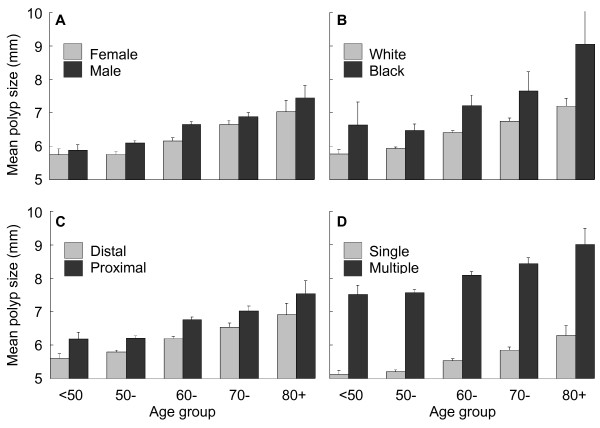
**Age-related differences in mean polyp size and 95% confidence intervals in relation to gender (A), race (B), location in colon (C), and single versus multiple polyps (D)**. Upper limit for 95% confidence limit for Blacks was 11.16.

Table 2 and Figure 1 demonstrate that for all patients and all subgroups, mean polyp size increases steadily with age. In all age groups, polyps were larger in males than in females, in Blacks than in Whites, proximal polyps were larger than distal polyps, and multiple polyps were larger than single polyps. (*P *< .0001) Overall, mean polyp size was larger in polyps removed in a Community or HMO site than in other sites, but the results were not consistent across all age groups. The presence or absence of a positive family history for bowel cancer did not appear to influence age-related changes in mean polyp size.

**Table 2:** Variables associated with mean size of largest polyp (mm) in each age group of patients.*

	Age Group				
Variable	< 50 n = 3097	50-59 n = 22859	60-69 n = 14913	70-79 n = 6991	80 + n = 1087
**Gender**					
Male	5.88 (5.72-6.0)**	6.1 (6.04-6.16)	6.64 (6.56-6.72)	6.88 (6.76-7.00)	7.45 (7.08-7.82)
Female	5.74 (5.56-5.92)	5.76 (5.69-5.83)	6.15 (6.06-6.24)	6.64 (6.51-6.77)	7.03 (6.68-7.38)
**Site**					
Academic	5.43 (5.07-5.79)	5.62 (5.47-5.77)	6.03 (5.83-6.23)	6.18 (5.92-6.44)	6.70 (5.83-7.57)
Community/HMO	5.88 (5.75-6.01)	5.99 (5.94-6.04)	6.51 (6.44-6.58)	6.87 (6.77-6.97)	7.27 (7.00-7.54)
VA/Military	5.68 (5.28-6.08)	5.98 (5.85-6.11)	6.28 (6.12-6.44)	6.60 (6.27-6.93)	7.60 (6.37-8.83)
**Race**					
White	5.77 (5.65-5.89)	5.93 (5.89-5.97)	6.40 (6.34-6.46)	6.74 (6.65-6.83)	7.19 (6.94-7.44)
Black	6.63 (5.95-7.31)	6.46 (6.26-6.66)	7.21 (6.89-7.53)	7.66 (7.09-8.23)	9.05 (6.94-11.16)
**Polyp location**					
Distal	5.60 (5.46-5.74)	5.79 (5.73-5.85)	6.19 (6.12-6.26)	6.54 (6.42-6.66)	6.91 (6.58-7.24)
Proximal	6.18 (5.98-6.38)	6.20 (6.13-6.27)	6.75 (6.66-6.84)	7.02 (6.88-7.16)	7.55 (7.17-7.93)
**Number of polyps**					
Single	5.12 (5.00-5.24)	5.21 (5.16-5.26)	5.53 (5.47-5.59)	5.85 (5.75-5.95)	6.29 (6.01-6.57)
Multiple	7.52 (7.25-7.79)	7.58 (7.49-7.67)	8.10 (7.99-8.21)	8.44 (8.27-8.61)	9.02 (8.54-9.50)
**Family history**					
Negative	5.80 (5.53-6.07)	5.93 (5.88-5.98)	6.43 (6.36-6.50)	6.84 (6.74-6.94)	7.29 (7.01-7.57)
Positive	5.82 (5.69-5.89)	6.07 (5.97-6.17)	6.50 (6.37-6.63)	6.49 (6.29-6.69)	7.04 (6.47-7.61)
**All patients**	5.82 (5.70-5.94)	5.96 (5.92-6.00)	6.44 (6.38-6.50)	6.78 (6.69-6.87)	7.25 (7.00-7.50)

*Group data are based upon averages of the single largest polyp in millimeters detected in each individual patient in that group. **Numbers in brackets = 95% confidence interval.

Table 3 lists the separate results of multivariate ordinal logistic regression for males and females. In general, the results resemble the findings resulting from the size/age analysis using mean polyp size, but the results are adjusted for all other study variables. The number of polyps found at colonoscopy (1, ≥ 2) proved to be the strongest predictor of age-related increase in polyp size.

**Table 3:** Multivariate ordinal logistic regression analysis of risk factors associated with polyp size in males and females

		Males (n = 48,395)		Females (n = 34,246)	
		OR	(95% CI)	OR	(95% CI)
**Age**					
	< 50	1.00		1.00	
	50 - 59	1.06	(0.98 - 1.16)	1.00	(0.91 - 1.11)
	60 - 69	1.29	(1.18 - 1.40)**	1.14	(1.03 - 1.26)*
	70 - 79	1.36	(1.24 - 1.49)**	1.34	(1.20 - 1.49)**
	80+	1.55	(1.35 - 1.79)**	1.46	(1.25 - 1.70)**
**Site**					
	Academic	1.00		1.00	
	Community/HMO	1.26	(1.18 - 1.34)**	1.22	(1.13 - 1.33)**
	VA/Military	0.97	(0.90 - 1.05)	0.90	(0.78 - 1.03)
**Race**					
	White	1.00		1.00	
	Black	1.31	(1.21 - 1.42)**	1.36	(1.24 - 1.48)**
**Location**					
	Distal Colon and rectum	1.00		1.00	
	Proximal Colon	1.11	(1.08-1.15)**	1.33	(1.27-1.39)**
**Number of polyps**					
	Single	1.00		1.00	
	Multiple	3.41	(3.29 - 3.54)**	3.04	(2.90 - 3.18)**
**Family history**					
	Negative	1.00		1.00	
	Positive	1.00	(0.95 - 1.05)	1.03	(0.98 - 1.08)

All odds ratios are adjusted for all other covariates in the table. * *P *< .01 **P < .0001

Odds Ratios (OR) and 95% Confidence Intervals (CI) obtained from ordinal logistic regression. Polyp size was stratified into 4 categories: 0-5, > 5-9, > 9-15, and > 15-40 mm. The odds ratio is the risk associated with an increase of polyp size corresponding to moving from one category to the next larger category, i.e. ≤ 5 mm vs. > 5 mm; ≤ 9 vs. > 9; ≤ 15 vs. > 15 mm.

## Discussion

Only a few previous studies have looked at polyp growth rates based on observed changes over time in patients with unresected polyps. In an early study, Welin and co-workers estimated the growth of tumors and colorectal polyps based on change of observed size in patients with multiple barium enema studies[6]. The study included 17 patients with adenomatous polyps who were followed for up to ten years after the initial polyp detection. The authors concluded that growth rates were similar for pathologically proven polyps and cancer, that growth rates were generally slow even for cancers, and that it was impossible to distinguish between linear and exponential growth rates.

After the advent of colonoscopy, a few follow-up studies of polyps detected and marked at the time of the initial screening examination have been performed. Hofsted and co-workers studied 58 patients over a three year period and found that 40% of polyps became larger, 27% were unchanged, and in 35% there was an apparent reduction in size[7]. Approximately half of the polyps included in the study were ≤ 4mm, so that measuring exact change in size would have been problematic. In a more recent study where 26 patients with marked polyps were followed over a period of two years, the findings were similar with inconsistent growth rates, and again, some polyps appeared to diminish in size during the study period[12]. Kozo, in a study of 33 patients enrolled in the placebo arm of a therapeutic trial found that polyp size increased about 5% per year but with wide variation[13].

These comparatively small studies imply that polyp growth rates are inconsistent and that in some cases polyps diminish in size over time. Data from a modeling exercise based on the national polyp data postulated that a regression in polyp size could explain the discrepancy between the large number of polyps in the population and the observed incidence of colorectal cancer[14].

In our study, mean differences in polyp size were present for study variables in most age groups and were initially observed in the youngest patient groups. Size differences were particularly striking for the number of polyps: in both univariate and multivariate analysis multiplicity of polyps, present in about one-third of patients, was the strongest risk factor for polyp size. The size of the largest polyp in patients with multiple polyps was approximately 50% larger than the largest polyp found in patients with only a single polyp. This finding suggests that either the growth pattern of multiple polyps is more aggressive than single polyps, or that initial polyp formation in patients with multiple polyps begins considerably earlier than in patients with a single polyp. This finding provides strong evidence supporting the recommendation that the screening interval in patients with multiple polyps be shorter than for patients with single polyps[15].

This study confirms and extends the previously reported findings from the CORI database relating to age and race as determinants of polyp size[9,16]. In both univariate and multivariate analysis, these two variables were strongly associated with polyp size in all age groups. In addition, gender, polyp location, and number of polyps were also found to be associated with polyp size.

Information about life style factors are not routinely collected in the CORI data set. Polyp formation has been related to factors such as smoking, alcohol and obesity and these factors could be important for polyp growth[17-21]. Additional studies need to be performed to determine the relationship between factors that predict polyp size reported here and environmental and genetic factors. Factors such as age, gender, polyp location and polyp multiplicity along with environmental factors are likely to affect mutational rates of crypt cells causing differences in the growth rates of polyps. Genetic factors which lead to genetic instability and which affect the growth rate of precursors of colorectal cancer undoubtedly play an important role[22,23]. Smoking and alcohol drinking are more prevalent in males and might explain the male/female differences in polyp size. It is of interest that a recent report based on colonoscopy screening in a different cohort found that smoking was significantly related to polyp size[24].

Family history of bowel cancer was not a predictor of polyp size in our data set. Our finding agrees with results from a smaller study based on the same CORI data where pathologic data were indicated that a positive family history for bowel cancer was not associated with an increased proportion of patients with advanced neoplasia[4]. Our results about family history contrast with the results of a direct observational study of polyp growth, where family history appeared to be a risk factor for polyp growth[10]. However this study was based on only 14 patients with a positive family history of bowel cancer who had been enrolled in the placebo arm of a randomized trial. In a follow-up study of patients after an initial screening colonoscopy, Nusko and co-workers found that a positive family history of colorectal cancer increased the risk of developing a metachronous tubular adenoma[11].

The strengths of this study relate to the large number of colonoscopies performed in various settings, with uniformity of data collection maintained by using a single reporting form. The procedures were carried out in two different racial groups in a variety of settings, so that the findings are likely to be valid for the general population.

This study has several weaknesses. In particular, because pathologic information was unavailable for patients from three-quarters of the centers, we did not include this variable in the analysis. Restricting the data set to those patients with a confirmed pathologic diagnosis would have greatly reduced the power of the study. By setting the upper limit of polyp size at 4 cm, it is likely we have captured all polyps detected at screening colonoscopy, but predictably, the data set includes patients with advanced histology. A previous report provides pathologic information based on nearly 6,000 patients included in the CORI database undergoing screening colonoscopy where pathologic information was available [4]. Based on this report, we estimate that about 8% of polyps in the current study would have been classified as having advanced histology including cancer, 51% a tubular adenoma, and 41% of patients would have a non-neoplastic (hyperplastic, inflammatory, lymphoid tissue) lesion.

Although the results are based on the initial screening at a participating center, it is possible that some patients may have had a previous examination in a non-participating center prior to being examined at a participating center. It is possible that patients with a positive family history, and those patients visiting academic, VA or military centers, would be more likely to have undergone prior examinations and that black patients might have been less likely to have had a prior colonoscopy. If so, then this differential exposure to a prior screening colonoscopy could partially explain some of our findings.

We performed multivariate analysis using available information. However we were unable to adjust for either lifestyle factors or genetic factors; if available these additional factors might have altered our findings.

Another study weakness is that there is likely to be measurement error of recorded polyp size leading to misclassification of polyp size. We believe this measurement error is likely to be bi-directional and based upon a previous sensitivity analysis of polyp size in CORI data, is unlikely to invalidate the findings. The analysis was limited to patients who underwent a first colonoscopy at a CORI-participating site during the study period, but we do not have information on whether the procedure was the patient's first ever, or just their first in the study period. Although this adds uncertainty about what we are measuring (a small polyp or lack of any polyp could occur in a patient who had prior polypectomy), this would only serve to underestimate our findings, as older patients are certainly more likely to have had prior screening compared to younger patients. A final weakness limiting our ability to study patient-specific growth patterns is that we analyzed aggregate patient data. The ideal method for studying polyp growth would be to use serial measurements over time in individual patients. But as pointed out previously, because of ethical considerations, such studies have rarely been performed.

In this study we studied the size of the largest polyp detected at screening colonoscopy performed in a large sample of patients in various practice settings. Although it is impossible to make exact inferences about polyp growth rates from our cross-sectional data, the observed age-related changes in mean polyp size serve as a surrogate measure of polyp growth. In our study younger patients are likely to have smaller polyps and there appears to be a linear relationship between age and polyp size. Overall polyp size increased gradually with age, and other factors such as gender, race, location of polyp, and the number of polyps were strong independent predictors of polyp size. Our data suggest that for many patients, several decades must elapse before a polyp initially measuring ≥ 5 mm attains a size where there is a significant risk of advanced pathology.

Decision models of colorectal screening make assumptions about polyp dwell time to help determine intervals between examinations. These data provide quantitative information about polyp duration which may prove useful for future decision models. The data also includes information about other variables that might help clinicians and policy makers determine the timing of initial colonoscopy, the frequency of follow-up procedures, and the most suitable screening procedure for individual patients.

## Conclusions

Our results indicate that polyp size is related to several factors including race, and gender and that there appears to be a nearly linear relationship between polyp size and age. The strongest risk factor for increasing polyp size over time was the presence of multiple polyps. Our findings suggest that small polyps will require several decades before attaining clinically significant size. The study augments existing information about determinants of polyp size and can help adjust screening recommendations based upon individual patient parameters.

## Competing interests

There are no competing interests. Dr. Lieberman is the executive director of the Clinical Outcomes Research Initiative (CORI), a non-profit organization that receives funding from federal and industry sources. This potential conflict of interest has been reviewed and managed by the Oregon Health Science University and the Portland Veterans Administration Conflict of Interest in Research Committees.

## Authors' contributions

ABL initiated the study, participated in the statistical analysis, and drafted the manuscript. JLW performed the statistical analysis, and assisted in preparing the manuscript. JLB participated in the study design, statistical analysis, and in preparing the manuscript. PM aided in study design and in manuscript preparation. DAL was responsible for data integrity, provided conceptual insight, assisted in data accrual, and participated in manuscript preparation. All authors read and approved the final manuscript.

## Pre-publication history

The pre-publication history for this paper can be accessed here:

http://www.biomedcentral.com/1471-230X/11/101/prepub
